# Primary gastric tuberculosis mimicking gastric lymphoma: a case report and multimodality imaging findings

**DOI:** 10.3389/fmed.2026.1956199

**Published:** 2026-10-07

**Authors:** Zhengguo Wan, Yuanyuan Sun, Penghua Liu, Wen Jia

**Affiliations:** CT and MRI Department, Handan First Hospital, Handan, Hebei, China

**Keywords:** computed tomography, gastric lymphoma, gastric tuberculosis, magnetic resonance imaging, primary gastric tuberculosis

## Abstract

Gastric tuberculosis represents a rare form of extrapulmonary tuberculosis. Owing to its non-specific clinical presentation, the condition is highly prone to misdiagnosis. It can mimic peptic ulcer disease or malignancy, typically manifesting as ulcerative lesions or gastric outlet obstruction, whereas less frequent presentations include massive gastrointestinal haemorrhage and gastric perforation. Consequently, establishing a definitive diagnosis remains challenging. This case report aims to augment the current understanding of this entity. Herein, we describe a 50-year-old female who presented with unexplained epigastric pain persisting for over nine months. Despite having undergone two prior gastroscopic examinations, the aetiology remained elusive and her symptoms persisted. Endoscopy revealed superficial gastritis and a submucosal lesion in the gastric body. Subsequent evaluation at our institution using endoscopic ultrasonography (EUS), computed tomography (CT), and magnetic resonance imaging (MRI) demonstrated gastric wall thickening, initially suggestive of gastric lymphoma. However, EUS-guided biopsy of the posterior gastric wall, with histopathological examination via haematoxylin and eosin (H&E) staining, disclosed chronically active mucosa with necrotizing granulomatous inflammation, numerous multinucleated giant cells, and occasional acid-fast bacilli (AFB), leading to a diagnosis of gastric tuberculosis. Given the non-specific symptomatology and imaging features that differ only subtly from those of gastric adenocarcinoma, lymphoma, and other submucosal tumours, the definitive diagnosis of gastric tuberculosis relies primarily on histopathological and immunohistochemical evaluation. By summarizing the imaging characteristics observed in this case, this report seeks to heighten clinical awareness and facilitate the recognition of this rare condition.

## Introduction

Extrapulmonary tuberculosis accounts for approximately 12% of global tuberculosis cases. Within this, gastrointestinal tuberculosis constitutes 11%–16% of extrapulmonary cases, representing 1%–3% of the total tuberculosis burden. Among gastrointestinal tuberculosis presentations, the ileocecal region is the most commonly affected site. In contrast, gastric tuberculosis remains rarely reported (1). Whether primary or secondary, the incidence of gastric tuberculosis in routine autopsy reports ranges from 0.03% to 0.21% (2). Clinically, gastric tuberculosis often mimics or coexists with peptic ulcers, carcinoma, or lymphoma under endoscopic examination (3). Its clinical presentation, whether primary or secondary infection, is often non-specific. Consequently, diagnosing gastric tuberculosis is highly challenging and frequently leads to misdiagnosis. Particularly when compared to other forms of tuberculosis, gastric tuberculosis presenting as a submucosal tumor-like lesion is more susceptible to misdiagnosis as lymphoma or gastrointestinal stromal tumor (GIST). We report a case of gastric tuberculosis that was initially misdiagnosed as lymphoma. The case data and imaging findings are provided below to enhance understanding of this disease. Contrast-enhanced abdominal CT revealed localized thickening along the lesser curvature of the gastric body, which appeared heterogeneously slightly hypodense on non-contrast scans. On post-contrast imaging, the peripheral zone of the lesion demonstrated marked persistent ring-like enhancement, while the central portion showed heterogeneous mild-to-moderate enhancement, accompanied by multiple enlarged perigastric lymph nodes. The patient was referred to our hospital in August 2022 for further evaluation and treatment.

On admission, the physical examination showed: body temperature of 35.8 ℃, respiratory rate of 17 breaths/min, heart rate of 77 beats/min, and blood pressure of 130/76 mmHg. Normal facial appearance, no signs of emaciation, good nutrition, no jaundice in skin or sclera, and no palpable superficial lymphadenopathy. Heart rhythm was regular. Breath sounds were clear bilaterally. Abdomen was flat without visible intestinal pattern or peristaltic waves, soft with no palpable masses, no tenderness or rebound tenderness. Liver and spleen were non-palpable below the costal margin. Murphy's sign was negative, no shifting dullness. Bowel sounds were normoactive at 4 per minute. Past history of cesarean section. No history of infectious diseases (e.g., tuberculosis, hepatitis) or chronic conditions (e.g., diabetes, hypertension). No known drug or food allergies.

### Laboratory findings

Complete Blood Count (CBC): Eosinophils: 9.3% (reference range: 0.4–8.0), mean Corpuscular Volume (MCV): 81 fL (reference range: 82–100), Mean Corpuscular Hemoglobin (MCH): 26.2 pg (reference range: 27–34), Erythrocyte Sedimentation Rate (ESR): 23 mm/hr (reference range: 0–20). Liver Function and Biochemistry:Alkaline Phosphatase (ALP): 138 U/L (reference range: 50–135), Albumin/Globulin Ratio (A/G): 1.2 (reference range: 1.0–2.4).

Immunological Profile: Immunoglobulin G (IgG): 17.10 g/L (reference range: 7.51–15.60), Immunoglobulin M (IgM): 3.13 g/L (reference range: 0.46–3.04), Anti-Nuclear Antibody (ANA): Positive-Anti-SS-A/Ro52 Antibodies: SS-A (+),Ro52 (+++). Tumor Marker: Neuron-Specific Enolase (NSE): 16.47 ng/mL. Peripheral blood T-cell assay for tuberculosis infection: T-N15 (reference range <6), antigen well T15 (reference range <6). The T-SPOT.TB test was positive, indicating a positive result of the tuberculosis interferon-gamma release assay. Tuberculosis antibody (TBAB) test was negative.

### Auxiliary examination

August 16, 2022, Chest CT findings: Bilateral pulmonary fibrous proliferative foci. Contrast-enhanced abdominal CT performed on July 19, 2022, demonstrated focal thickening of the gastric wall along the lesser curvature. On non-contrast images, the lesion appeared heterogeneously slightly hypodense. Post-contrast imaging revealed marked persistent ring-like enhancement in the peripheral zone, with heterogeneous mild-to-moderate enhancement in the central portion (Figure 1A–C). Multiple enlarged lymph nodes were observed in the hepatogastric space, raising suspicion for metastatic involvement. On August 17, 2022, unenhanced gastric MRI revealed focal thickening of the lesser curvature of the stomach. The lesion appeared slightly hyperintense on T1-weighted imaging (T1WI), slightly hyperintense on T2-weighted fat-suppressed sequences, hyperintense on high-b-value diffusion-weighted imaging (DWI), and hypointense on the apparent diffusion coefficient (ADC) map. Multiple enlarged lymph nodes observed in liver-gastric space, showed slightly hyperintense on T1WI fat-suppressed sequence; reverse position signal slightly higher than same-direction position signal (Figure 2B–H). Based on the above findings, lymphoma cannot be excluded. EUS was performed in the afternoon of the same day. Gastric body examination: a 1.2 cm protruding lesion was identified on the posterior wall of the upper gastric body, with two patches of white mucus covering the lesion surface. The lesion exhibited well-demarcated borders and mixed echogenicity predominantly hypoechoic, measuring approximately 1.45 × 0.75 cm, raising a tentative suspicion of abscess. The lesion felt slightly soft during biopsy manipulation; tissue specimens were forceps-biopsied (Figure 2A, I). Thick white viscous fluid drained after irrigation, and histopathological analysis was pending. Scar-like changes were also noted on the posterior wall of the upper gastric body. Additional endoscopic findings included chronic non-atrophic gastritis with mucosal erosion.

**Figure 1:**
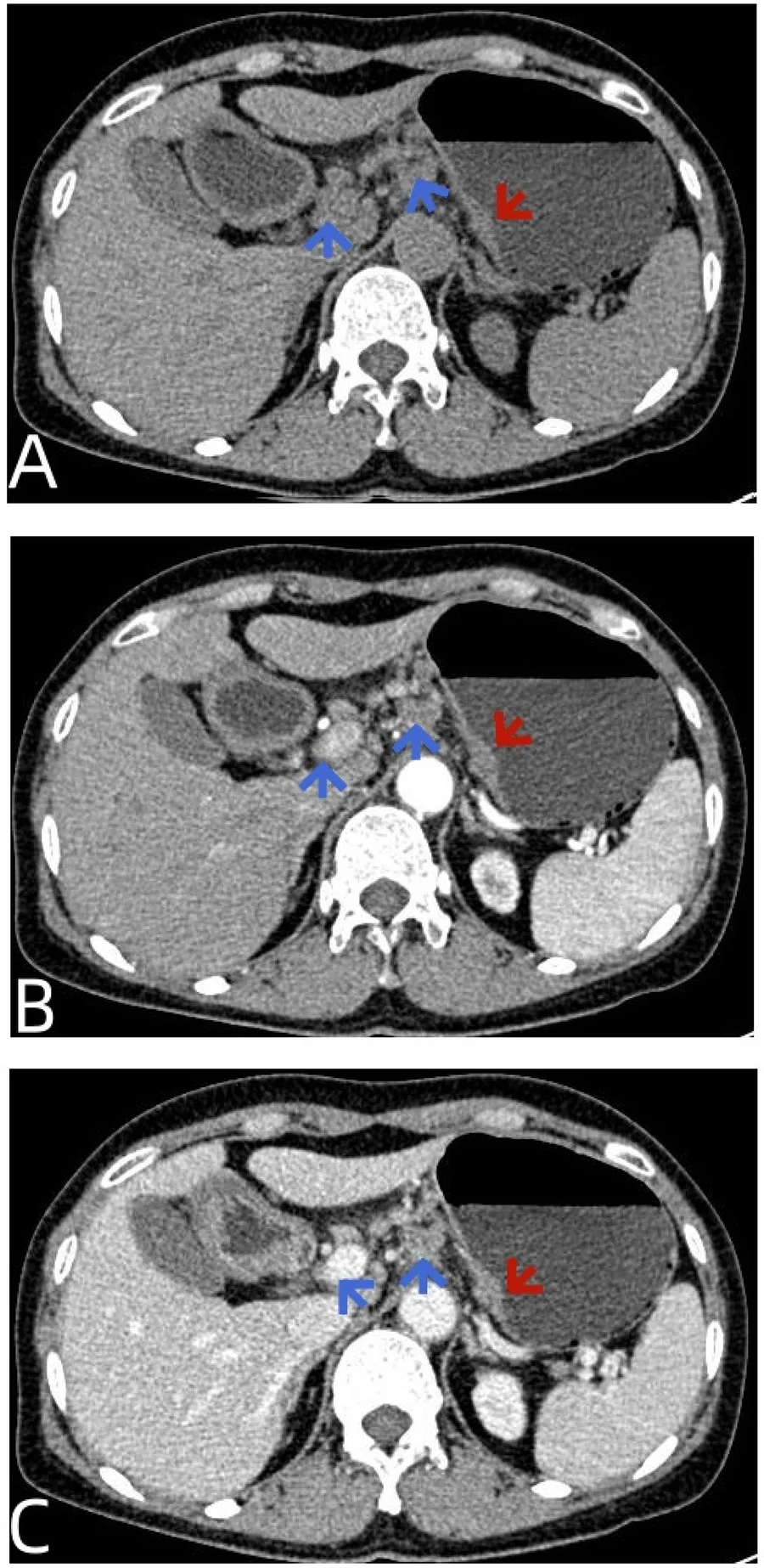
**(A)** Non-contrast CT shows gastric wall thickening (arrow) and perigastric lymphadenopathy. **(B,C)** Contrast-enhanced CT revealed marked persistent ring-like enhancement in the peripheral zone of the lesion, with heterogeneous mild-to-moderate enhancement in the central portion. Blue arrow, Lymph Node; Red arrow, Gastric lesion.

**Figure 2:**
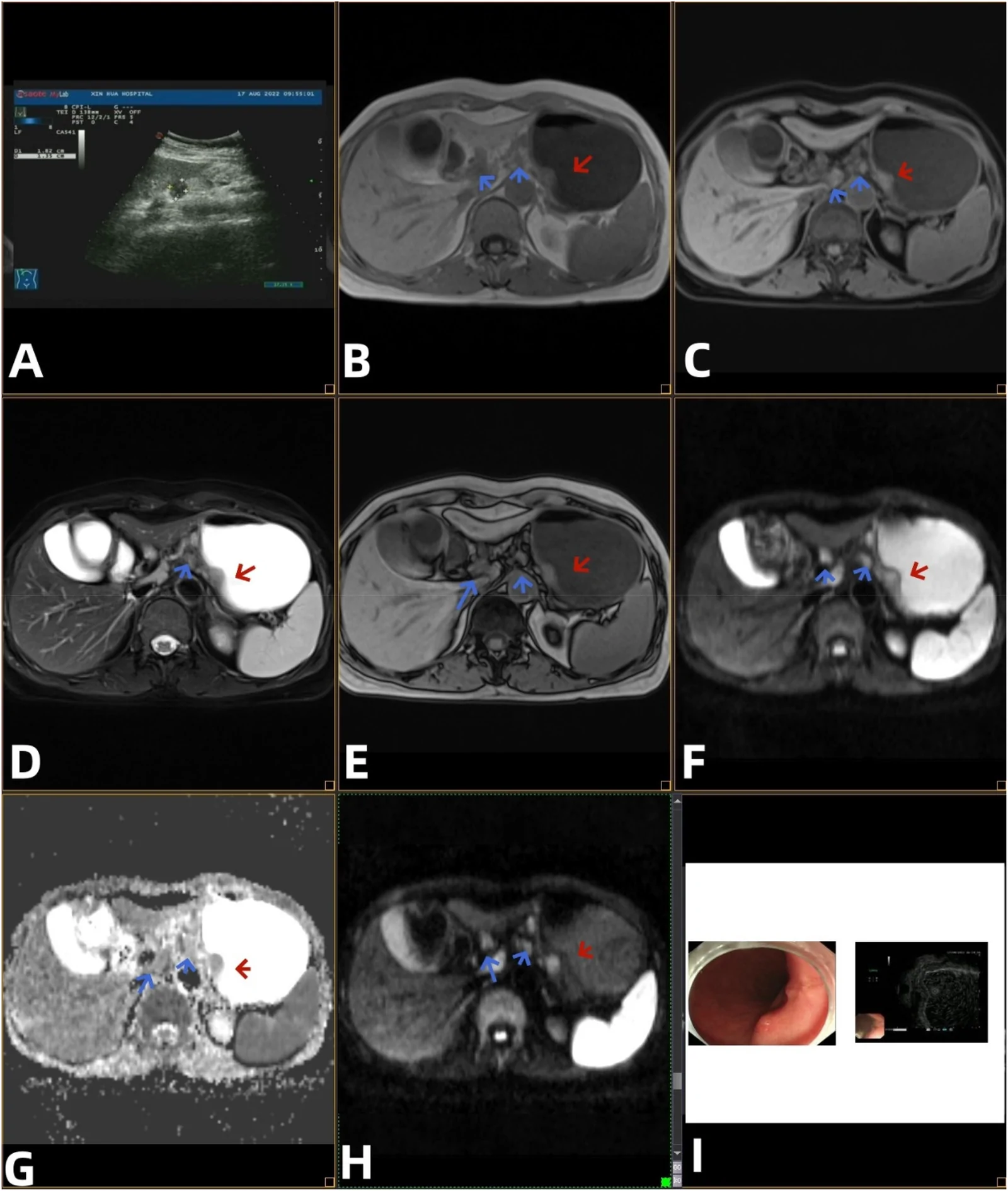
MRI Findings. **(A)** Ultrasound revealed a hypoechoic lesion. **(B)** In-phase T1-weighted imaging (T1WI) demonstrated gastric wall thickening with a slightly hypointense signal. Enlarged surrounding lymph nodes also exhibited mild hypointensity. **(C)** On fat-suppressed T1WI, both the gastric lesion and the lymph nodes appeared slightly hyperintense. **(D)** T2-weighted imaging (T2WI) showed thickened gastric wall and enlarged surrounding lymph nodes, both demonstrating slight hyperintensity. *These imaging features are suggestive of granulomatous inflammation, consistent with gastric tuberculosis. **(E)** Opposed-phase T1-weighted imaging (T1WI) demonstrated mildly increased signal intensity within the gastric peripyloric lymph nodes compared to the in-phase sequence. **(F–H)** On diffusion-weighted imaging (DWI) with high b-value, both the gastric wall lesion and the surrounding lymph nodes appeared hyperintense and demonstrated restricted diffusion. **(I)** EUS revealed a 1.2 cm elevated lesion in the upper gastric body. The lesion appeared heterogeneously hypoechoic, was soft on pressure applied by biopsy forceps, and released whitish mucus upon water irrigation. Adherent whitish mucus was also noted on the lesion surface. Blue arrow, Lymph Node; Red arrow, Gastric lesion.

Histopathological examination of the single biopsy specimen harvested from the posterior gastric wall demonstrated moderately active chronic mucosal inflammation complicated by necrotizing granulomatous inflammation, abundant multinucleated giant cells, and rare acid-fast bacilli; Helicobacter pylori (Hp) testing yielded negative results. Interferon-gamma release assay (IGRA) for peripheral blood tuberculosis infection showed T-N value of 15 (reference range <6) and antigen-stimulated T-cell count of 15 (reference range <6). The patient had no prior history of pulmonary or extrapulmonary tuberculosis, and a final diagnosis of primary gastric tuberculosis was established.

The treatment plan includes a four-drug anti-tuberculosis regimen: Oral Isoniazid (5 mg/kg once daily), Rifampicin (10 mg/kg once daily), Ethambutol (15 mg/kg once daily), and Pyrazinamide (25 mg/kg once daily) for 6 months. Supportive therapies, such as liver protection, gastric mucosal preservation, and immune regulation, are also being administered. The patient received a standard 6-month anti-tuberculosis regimen, which is guideline-recommended for uncomplicated, drug-susceptible extrapulmonary tuberculosis to ensure cure and avoid overtreatment. Regular clinical and laboratory monitoring including liver and renal function tests and blood cell counts was performed throughout treatment. The patient showed good adherence and completed the full therapeutic course. At the 12-month follow-up, the patient reported marked improvement in clinical symptoms, with complete resolution of abdominal complaints. Follow-up CT imaging confirmed no recurrence of gastric tuberculosis, and the previously noted lymph nodes had decreased in size.

## Discussion

Gastric tuberculosis is a rare clinical entity first described in 1,842 (4). Globally, extrapulmonary tuberculosis (EPTB) is estimated to account for 8%–24% of tuberculosis (TB) cases, with a mean of 15% of all TB cases notified to WHO (5). In China, EPTB accounts for approximately 31% of all TB cases. Among these EPTB cases, the skeletal system was unexpectedly the most common site (41%), followed by the pleura (26%). Abdominal sites were not specifically assessed, and other unclassified sites accounted for 14% of all EPTB cases (6). In Europe, extrapulmonary sites account for 17% of all TB cases, with the abdomen accounting for approximately 3%, making it the sixth most common site (7). In a multicentre cohort of co-infected patients, EPTB accounted for 28% of all TB cases, and abdominal TB ranked third among affected sites (8).The incidence of TB is highest on the Asian and African continents and is also rising sharply in developed countries, driven primarily by latent tuberculosis infection (LTBI) resulting from immunocompromising conditions, the use of biological agents, and population migration. Gastric tuberculosis represents an extremely rare subtype of abdominal TB, which easily mimics gastric malignancy or peptic ulcer and is often misdiagnosed (5). Approximately 30% of the global population is latently infected with TB, of whom 10%–15% may reactivate to progress to active tuberculosis (5). Use of anti-tumor necrosis factor (TNF)-*α* agents. TNF-α serves as a critical mediator of the human immune response against tuberculosis infection; reduced activity increases susceptibility to infection. Accordingly, administration of anti-TNF-α agents is recognized to reactivate LTBI (9). In a meta-analysis of tuberculosis among liver transplant recipients, abdominal tuberculosis represented the most common site of involvement, accounting for 35% of EPTB cases. In a multicenter study of tuberculosis in renal transplant recipients, abdominal tuberculosis was the predominant site, constituting 30% of EPTB manifestations (10, 11). The pathogenesis of extrapulmonary tuberculosis remains incompletely elucidated. Multiple factors governing pathogen–host interactions may contribute to disease development. Impaired immune function constitutes a risk factor for extrapulmonary tuberculosis (12). Our patient exhibited immune compromise, and gastroscopic biopsy confirmed chronic non-atrophic gastritis with erosion. These factors may have facilitated the development of gastric tuberculosis in this patient.

Gastric tuberculosis may arise through the following pathways (3, 13, 14): 1) Direct mucosal invasion: Mycobacterium tuberculosis from ingested sputum or contaminated food invades the gastric epithelium. Compromised mucosal integrity enables deeper penetration into submucosal, muscular, or serosal layers, leading to granuloma formation. 2) Hematogenous/lymphatic spread: Dissemination via blood or lymphatics seeds bacilli within the gastric wall, often affecting submucosal and muscular layers before involving mucosa or serosa. 3) Contiguous extension: Direct invasion from nearby infected sites, such as tuberculous peritonitis or adjacent organs. 4) Secondary superinfection: Preexisting gastric lesions-such as ulcers or carcinomas-disrupt the mucosal barrier, reduce acidity, and facilitate mycobacterial colonization. Our patient had no history of extrapulmonary tuberculosis. On evaluation, she was found to have underlying immune system impairment. Gastroscopic biopsy confirmed chronic non-atrophic gastritis with erosions. In the context of compromised immune function, the bacteria likely gained entry through the disrupted mucosal barrier and established primary foci. We therefore postulate that the route of infection in this case was most probably direct invasion of the gastric mucosa by Mycobacterium tuberculosis. Gastric involvement is highly uncommon and typically localizes to the antrum or cardia. This condition usually occurs secondary to active tuberculosis elsewhere-most commonly pulmonary-or in immunocompromised patients. The stomach possesses natural defense mechanisms, such as bactericidal gastric acid, minimal lymphoid tissue, and an intact mucosal barrier, that confer inherent resistance to Mycobacterium tuberculosis colonization. The precise pathogenesis remains unclear, but contributing factors likely include low lymphatic density in the gastric wall, acidic environment, efficient motility, and mucosal integrity (3, 4).When these protective mechanisms are compromised-through reduced acid secretion, impaired motility, or mucosal injury-bacterial persistence and infection risk increase. The gastric antrum is particularly susceptible due to its relatively abundant lymphoid tissue, which provides a suitable environment for mycobacterial proliferation given the pathogen's affinity for lymphatic tissue (15, 16).

The clinical presentation of gastric tuberculosis is nonspecific, often resembling peptic ulcer disease, gastric carcinoma, or lymphoma. Epigastric pain is the most common symptom. Some patients develop gastric outlet obstruction or palpable epigastric masses; gastrointestinal bleeding is uncommon. Endoscopic findings usually show atypical gastroduodenal ulcers or malignant-appearing lesions. In a review of 49 cases of duodenal tuberculosis by Gleason et al. (17), pain (73%) and vomiting (55%) were predominant, whereas bleeding was rare (16%). This nonspecificity often leads to misdiagnosis. Despite comprehensive evaluation with endoscopy, EUS, and abdominal CT, preoperative diagnosis is seldom achieved. The ambiguous nature of the clinical presentation frequently leads to misdiagnosis. Moreover, owing to the lack of a comprehensive understanding of the imaging features of gastric tuberculosis, achieving a definitive preoperative diagnosis remains challenging.

Gastric tuberculosis is classified into four types based on anatomical extent and comorbidity status (e.g., isolated miliary gastric TB without systemic dissemination): 1) Localized type: Most frequent, with tuberculous lesions confined to the gastric antrum or body; antral involvement may manifest as gastric outlet obstruction. 2) Ulcerative type: Predominant pattern, featuring single or multiple tuberculous ulcers exhibiting variable dimensions, irregular margins, and surrounding inflammatory or necrotic changes. 3) Diffuse type: Less common, involving contiguous structures (stomach, duodenum, pancreatic head) or perigastric lymph nodes, frequently associated with extensive abdominal lymphadenopathy. 4) Concomitant type: Rare co-occurrence with gastric carcinoma or peptic ulcer disease (3, 14, 18).

Imaging manifestations of gastric tuberculosis lack pathognomonic features (19, 20). This patient presented with epigastric pain and abnormal immune biomarkers, complicated by chronic gastritis and gastroduodenal ulcer. Primary pulmonary tuberculosis typically involves the upper lobes and dorsal segments of the lower lobes of both lungs, with variable morphologies including miliary, nodular, and patchy opacities, and cavitation may occur. In the present case, the bilateral pulmonary lesions were scattered, manifesting as linear opacities, tiny and small nodules with well-defined borders, which were inconsistent with the features of primary tuberculosis. The patient denied any history of tuberculosis exposure and exhibited no systemic constitutional symptoms including fatigue, low-grade fever or night sweats. On non-contrast CT, the lesion manifested as localized thickening of the gastric wall with heterogeneous slightly hypodense appearance. Post-contrast imaging demonstrated marked persistent ring-like enhancement in the peripheral zone and heterogeneous mild-to-moderate enhancement in the central portion. This pattern differed from the diffuse marked thickening typically seen in lymphoma, which usually shows progressive mild-to-moderate enhancement with an intact continuous mucosal layer, thereby essentially excluding early-stage carcinoma. MRI revealed restricted diffusion in the perigastric lymph nodes, which initially raised suspicion for lymphoma. EUS identified a well-circumscribed lesion with a surface covered by mucus and whitish secretions, exhibiting mixed echogenicity (predominantly hypoechoic), suggestive of a submucosal abscess. A definitive diagnosis of primary gastric tuberculosis was ultimately established based on a positive tuberculin skin test and histopathological demonstration of granulomatous inflammation. EUS findings were suggestive of a submucosal abscess. On T1-weighted fat-suppressed imaging, the lesion appeared slightly hyperintense, which is attributable to the fact that intermediate-sized molecules—such as those in granulomatous tissue—exhibit tumbling frequencies that match the Larmor frequency, thereby resulting in shortened T1 relaxation times. Consequently, the lesion demonstrates slight hyperintensity on T1-weighted fat-suppressed sequences. The surrounding lymph nodes similarly showed slight hyperintensity on T1-weighted fat-suppressed imaging, with signal intensity on out-of-phase imaging exceeding that on in-phase imaging (21, 22). This imaging profile distinguishes the lesion from early-stage carcinoma, leiomyoma, lymphoma, or GIST. Published literature has described the EUS features of gastric tuberculosis as homogeneous hypoechoic lesions arising from the muscularis propria of the gastric wall with well-demarcated margins. Endoscopic ultrasonography in our patient revealed a lesion covered by mucus with white secretions; the lesion had well-defined borders and exhibited mixed echogenicity predominantly hypoechoic (23). The diagnosis of primary gastric tuberculosis was ultimately confirmed by a positive tuberculin skin test and histopathological identification of granulomas.

Gastric lymphoma mainly involves the gastric body and antrum, presenting marked gastric wall thickening (>1 cm). On unenhanced CT, lesions show isoattenuation, with mild homogeneous enhancement after contrast administration. Mucosal involvement manifests as linear mucosal enhancement; low-attenuation necrotic foci may be present within the thickened gastric wall. Lymph node metastasis is common, and affected nodes demonstrate homogeneous enhancement. Lymph nodes exhibit no signal intensity change on T1-weighted in-phase/out-of-phase imaging (24). Gastrointestinal stromal tumors (GISTs) predominantly arise along the greater curvature of the gastric body, followed by the gastric fundus. On unenhanced CT, GISTs demonstrate isoattenuation and may contain necrosis, hemorrhage, or cystic degeneration. Lesions are typically round or oval; larger tumors can appear irregular or lobulated. Contrast-enhanced images show moderate or marked homogeneous enhancement, whereas heterogeneous enhancement occurs when central necrosis or cystic degeneration develops. Marked lymphadenopathy is rarely observed (25).Gastric carcinoma may arise in any gastric segment but is most prevalent in the gastric antrum. The affected gastric wall shows irregular thickening with convergence, disruption and destruction of mucosal folds. Lesions demonstrate heterogeneous, marked enhancement on contrast-enhanced imaging. In advanced-stage disease, the adjacent serosal surface becomes ill-defined, and regional lymph nodes frequently contain necrosis. These necrotic nodes exhibit low attenuation on unenhanced CT and heterogeneous rim enhancement after contrast administration (26). The unenhanced and contrast-enhanced CT findings in this patient overlapped with those of lymphoma. Previous MRI literature on gastric tuberculosis remains sparse, and the MRI manifestations of gastric tuberculosis are poorly characterized. Therefore, lymphoma could not be excluded in the initial differential diagnosis. Endoscopic ultrasonography suggested a possible abscess. Other granulomatous disorders, such as Crohn's disease and sarcoidosis, were also considered in the differential diagnosis. There were no clinical, endoscopic or histologic features supporting Crohn's disease (no transmural inflammation, no non-caseating granulomas, no skip lesions). Sarcoidosis was unlikely given the absence of systemic involvement and non-caseating granulomas. Fungal infection could not be definitively ruled out by special staining or fungal culture in this case, which is acknowledged as a limitation. Ultimately, histopathological examination of biopsied lesion tissue identified caseating granulomas, confirming gastric tuberculosis and excluding lymphoma, adenocarcinoma, GIST, as well as other granulomatous inflammatory disorders. Tuberculous lymph nodes typically exhibit caseous necrosis. The intermediate-sized protein-lipid complexes within the necrotic material alter the tumbling frequency of water molecules, thereby manifesting as slight hyperintensity on T1-weighted fat-suppressed sequences (21, 27). This imaging feature may aid in distinguishing tuberculous lymphadenopathy from lymphoma or metastatic carcinoma. In the present case, the restricted diffusion observed in the gastric tuberculous lesion was attributable to the dense cellular arrangement of granulomatous tissue with reduced extracellular space. Concurrently, the restricted diffusion in the surrounding lymph nodes resulted from the compact caseous necrotic material, which impeded water molecular motion. Although neoplastic lesions may show similar diffusion restriction due to densely packed tumour cells with markedly diminished extracellular space, the underlying histopathological substrate differs from that of tuberculosis (28).

Immunohistochemistry serves as an important adjunctive method for distinguishing gastric tuberculosis from gastric malignancies, though it was not performed in our patient. Epithelioid histiocytes in tuberculous granulomas are CD68-positive and cytokeratin-negative, excluding adenocarcinoma (29). CD20 and CD3 can differentiate reactive lymphocytes from clonal populations of gastric lymphoma, while CD117/DOG-1 staining helps rule out GIST. When morphology alone is inconclusive, this immunohistochemical panel helps discriminate infectious granulomatous disease from neoplasia (30).

Gastrointestinal tuberculosis is a paucibacillary form of tuberculosis; therefore, AFB may not be isolated from clinical specimens obtained via either non-invasive or invasive techniques (31). Although mycobacterial detection assays demonstrate high specificity in gastrointestinal tuberculosis, acid-fast staining, mycobacterial culture, and polymerase chain reaction (PCR) have been reported to show poor sensitivity (32). A meta-analysis of available studies demonstrated that the pooled sensitivity and specificity of PCR for AFB on endoscopic biopsy specimens were 44% and 95%, respectively, for distinguishing intestinal tuberculosis from Crohn's disease (33). A positive tuberculin skin test (TST) or interferon-gamma release assay (IGRA) result did not aid in the diagnosis, since a positive test does not necessarily indicate active disease (34). For patients with suspected gastric tuberculosis, non-contrast CT is the preferred first-line imaging modality for evaluating the location and extent of the disease. Contrast-enhanced CT, T1-weighted fat-suppressed MRI, in-phase and opposed-phase signal characteristics, DWI, and EUS can provide pathological insights into the cellular composition of the lesion. Definitive diagnosis relies on histopathological identification of caseating granulomas or AFB; mycobacterial PCR is considered the gold standard for diagnosis (23).

Gastrointestinal tuberculosis remains a major global health concern. Primary gastric tuberculosis has non-specific presentations and can affect any part of the gastrointestinal tract, occurring in both immunocompromised and immunocompetent individuals. Characteristic imaging features—including ring-like enhancement, restricted diffusion, and lymphadenopathy—should prompt consideration of gastric tuberculosis. Histopathological confirmation remains essential to differentiate gastric tuberculosis from other diseases. Definitive diagnosis requires multiple deep biopsies. Treatment follows standard anti-tuberculous regimens, with invasive measures reserved for complications.

## Patient perspective

Written informed consent was obtained from the patient for publication of this case report.

## Data Availability

The original contributions presented in the study are included in the article/Supplementary Material, further inquiries can be directed to the corresponding author.
